# Brief Report: Relationship Between Tumor Volume and Clinical Outcomes in Relapsed SCLC

**DOI:** 10.1016/j.jtocrr.2025.100904

**Published:** 2025-09-17

**Authors:** Matthew Lu, Hayden Byrd, Heidi Chen, Wade Iams

**Affiliations:** a Division of Hematology-Oncology, Department of Medicine, UT Southwestern Medical Center, Dallas, Texas; b Department of Radiation Oncology, Vanderbilt University Medical Center, Nashville, Tennessee; c Department of Biostatistics, Vanderbilt University Medical Center, Nashville, Tennessee; d Division of Hematology-Oncology, Department of Medicine, Vanderbilt University Medical Center, Nashville, Tennessee

**Keywords:** Clinical outcomes, Relapsed, Small cell lung cancer, Total body tumor volume

## Abstract

**Introduction:**

For patients with limited-stage SCLC (LS-SCLC), tumor burden is a predictor of clinical outcomes, but there are no known data exploring whether tumor burden is a predictor of clinical outcomes in relapsed SCLC.

**Methods:**

In this retrospective correlative study analyzing a cohort of 93 patients with relapsed SCLC, total body tumor volume at time of relapse (TV), progression-free survival (PFS), and overall survival (OS) were calculated and a Cox proportional hazards model was used to evaluate the relationship between TV and PFS and OS.

**Results:**

A total of 93 patients with relapsed SCLC were analyzed, of whom 70% initially had extensive-stage SCLC (ES-SCLC) and 30% initially had LS-SCLC. Eastern Cooperative Oncology Group performance status and receipt of second-line therapy were significantly associated with OS in a linear fashion (*p* = 0.002 and *p* = 0.0457, respectively). TV was significantly associated with OS in a nonlinear fashion, even when controlling for Eastern Cooperative Oncology Group performance status, response to initial therapy, chemotherapy-free interval, and receipt of second-line therapy (*p* = 0.0031).

**Conclusions:**

TV was observed to be a predictor of OS in patients with relapsed SCLC. Further studies are needed to evaluate whether initiation of standard systemic therapy at lower TV is able to consistently improve PFS and OS in relapsed SCLC.

## Introduction

SCLC is an aggressive cancer that currently accounts for 15% of lung cancers.^1^ At time of diagnosis, at least 80% of patients will have disease that has spread outside of the thoracic and supraclavicular area, which classifies them as extensive-stage disease, as opposed to limited-stage disease.^2^ Without treatment, median survival is 2 to 4 months.^1^ With treatment, clinical outcomes are still bleak with overall survival (OS) in limited-stage SCLC (LS-SCLC) being 16 to 24 months and 6 to 12 months in extensive-stage SCLC (ES-SCLC).^1^ SCLC is initially responsive to treatment, but unfortunately it often relapses within 6 to 12 months and is often resistant to the initial treatment.^3^ A considerable amount of research is currently focused on developing new combinations of treatments and novel targeted therapies in hopes of improving clinical outcomes.^4^

An additional avenue to improve clinical outcomes could be detecting and treating relapsed SCLC earlier, when tumor burden is lower, as prior research has found this to be the case in the initial treatment of LS-SCLC.^5^ Recent research reveals a correlation between tumor burden and blood-based circulating tumor DNA (ctDNA) assays, which presents an opportunity to more effectively and frequently screen for disease relapse.^6^ This study aimed to evaluate the relationship between tumor volume (TV) at time of first relapse and clinical outcomes in SCLC.

## Methods

Patients with relapsed SCLC were retrospectively identified from a Vanderbilt University Medical Center (VUMC) thoracic oncology database. Patients with relapsed SCLC were described as those with either initial LS-SCLC or ES-SCLC who received first-line therapy and subsequently experienced extracranial radiographic disease progression. Patients did not have to experience complete response from first-line therapy, nor did they have to receive second-line therapy after relapse to be included in the study. Patients who did not experience any improvement of disease in response to first-line therapy but still experienced disease progression were included in the study. This study was performed under an institutional review board–approved protocol, which included a waiver of informed consent. Date of relapse was assessed from review of clinical notes and radiology reports, not based on Response Evaluation Criteria in Solid Tumors (RECIST).

TV at time of first relapse was assessed using extracranial diagnostic computed tomography (CT) imaging or the CT portion of a positron emission tomography-CT (PET-CT) scan. Three-dimensional TV was calculated using MIM Software (Prod MIMpacs [10.100.216.19] MIM version 7.2.3 [Build M314-02]) by a radiation oncologist with the assistance of a formal radiology read. Tissue types involved with cancer were separately identified and reported in cubic centimeters (cc).

Progression-free survival (PFS) and OS were measured from the time of first relapse. PFS was defined as the time between the first relapse and the next date of progression. OS was defined as the time between the first relapse and death from any cause. Response to initial therapy was obtained from review of clinic notes and radiology reports and was classified as complete response, partial response, mixed response, stable disease, or progression of disease. Chemotherapy-free interval (CTFI) was defined as the time in days between end of first-line therapy or discontinuation of first-line therapy and the start date of second-line therapy. If a patient never received second-line therapy, then the date of progression or date of death was used instead to calculate the CTFI. If a patient received maintenance therapy, this was time was included in the CTFI. Receipt of second-line therapy was a binary variable, obtained from chart review of clinic notes. Cox proportional hazards (CPH) regression, which allows for evaluation of nonlinear effects using regression splines, was used to evaluate the association between TV and PFS and OS. If the *p* value of nonlinear TV effect was greater than 0.2, a linear TV effect was fit in CPH regression. When performing the multivariable CPH, Eastern Cooperative Oncology Group performance status (ECOG PS), TV, best response to initial therapy, CTFI, and receipt of second-line therapy were included in the analysis, as our intent to maximize statistical validity.

## Results

Between October 2014 and April 2024, 93 patients with relapsed SCLC and meeting the above inclusion criteria were treated at VUMC. Patients in the cohort had a median age of 72 years (80% ≥65 y old), were nearly equal in gender distribution, most reported as White (90%), and more likely to have ES-SCLC at time of diagnosis (70%).

In this cohort, 38% of patients with initial ES-SCLC received first-line therapy with carboplatin, etoposide and atezolizumab and 51% received carboplatin/etoposide. Of the patients with initial ES-SCLC, 83% received second-line therapy. Of these patients, 43% received immunotherapy, 20% received paclitaxel, 19% received lurbinectedin, and 17% received platinum doublet rechallenge. Furthermore, 40% of these patients received three or more lines of therapy.

In this cohort, 68% of patients with initial LS-SCLC received first-line therapy with cisplatin and etoposide and 32% received carboplatin and etoposide, with 92% of eligible patients receiving concurrent chemoradiotherapy (CCRT). Of patients with initial LS-SCLC, 82% received second-line therapy. Of these patients, 35% received platinum doublet rechallenge, 35% received immunotherapy, 17% received paclitaxel, and 13% received lurbinectedin. Furthermore, 68% of these patients received at least three lines of therapy (Table 1).

**Table 1:** Characteristics of 93 Patients With Relapsed SCLC

Characteristic	N = 93
Age, y, median (min–max)	72 (33–93)
Sex, n (%)	
Male	46 (49)
Female	47 (51)
Race, n (%)	
White	84 (90)
Black or African American	7 (8)
Asian	2 (2)
Ethnicity, n (%)	
Hispanic	1 (1)
Non-Hispanic	92 (99)
ECOG PS at time of diagnosis, n (%)	
0	17 (18)
1	59 (63)
2	9 (10)
3	8 (9)
Smoking status at time of diagnosis, n (%)	
Current	46 (50)
Former	42 (45)
Never	5 (5)
Stage at time of diagnosis, n (%)	
I	4 (4)
II	3 (3)
III	21 (23)
IV	65 (70)
Active CNS disease at time of diagnosis, n (%)	15 (16)
Tumor burden at time of relapse, n (%)	
Median, mean, mode (min–max)	68.3, 199.9, 122 (0.59–1927.6)
Less than 30 cc	22 (24)
30–60 cc	22 (24)
60–300 cc	36 (39)
Greater than 300 cc	13 (14)
First-line treatments received in ES-SCLC, n (%)	65 (100)
Carboplatin/Etoposide/Atezolizumab	25 (38)
Carboplatin/Etoposide	33 (51)
Cisplatin/Etoposide	4 (6)
Carboplatin/Irinotecan	3 (5)
Second-line treatments received in ES-SCLC, n (%)	54 (83)
Immunotherapy (Nivolumab ± Ipilimumab)	23 (43)
Paclitaxel	11 (20)
Lurbinectedin	10 (19)
Carboplatin/Etoposide	9 (17)
Clinical trial	1 (1)
3+ Lines of treatment in ES-SCLC, n (%)	26 (40)
First-line treatments received in LS-SCLC, n (%)	28 (100)
Cisplatin/Etoposide	19 (68)
Carboplatin/Etoposide	9 (32)
CCR received if indicated	23 (92)
Second-line treatments received in LS-SCLC, n (%)	23 (82)
Carboplatin/Etoposide	8 (35)
Immunotherapy (Nivolumab ± Ipilimumab)	8 (35)
Paclitaxel	4 (17)
Lurbinectedin	3 (13)
3+ Lines of treatment in LS-SCLC, n (%)	19 (68)
Other relevant data	
Patients enrolled in clinical trials at any time	13 (14)
Received any palliative radiation	41 (44)
Received consolidative thoracic radiotherapy	10 (11)
Received prophylactic cranial irradiation	25 (27)
CTFI ≥ 90 d	53 (57)
CTFI ≥ 6 mo	25 (27)
CT scans used to calculate TV at relapse	77 (83)
PET-CT used to calculate TV at relapse	16 (17)

CCRT, concurrent chemoradiation; CNS, central nervous system; CT, computed tomography; CTFI, chemotherapy-free interval; ECOG PS, Eastern Cooperative Oncology Group performance status; ES-SCLC, extensive-stage SCLC; LS-SCLC, limited-stage SCLC; PET-CT, positron emission tomography-computed tomography; TV, tumor volume.

Other relevant statistics of this cohort were as follows: 14% of patients were enrolled in a clinical trial as part of their treatment course, 44% received any type of palliative radiotherapy, 11% received consolidative thoracic radiotherapy, and 27% received prophylactic cranial irradiation (PCI). Of the patients, 57% had CTFI more than or equal to 90 days and 27% had CTFI more than or equal to 6 months. Furthermore, 83% of the scans used to calculate TV were diagnostic CTs, and the rest were PET-CTs.

Patients in the cohort received surveillance CT scans every 3 months on average. The range of TV was 0.59 cc to 1927.57 cc, with a mean TV of 199.91 cc and a median TV of 68.29 cc. Most of the patients had TV less than 300 cc (86%), and nearly most patients had a TV less than 60 cc (47%). There were equal amounts of patients with TV 0 to 30 cc and TV 30 to 60 cc (24% each).

**Figure 1:**
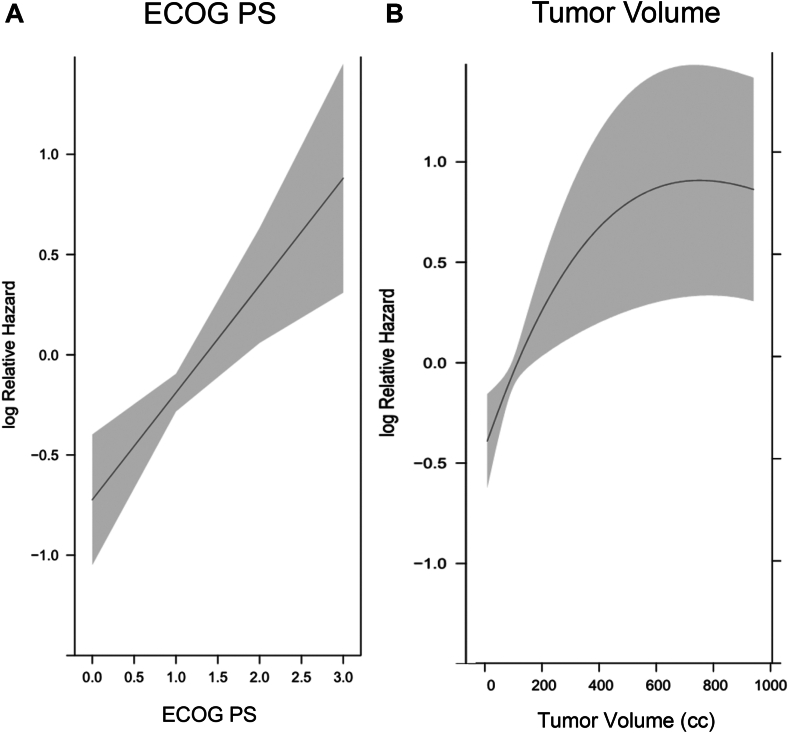
(*A*) Hazard for death versus ECOG PS. A Cox proportional hazard model revealed a statistically significant linear association between hazard for death and ECOG PS (*p* = 0.0003). (*B*) Hazard for death versus tumor volume. A Cox proportional hazard model revealed a statistically significant nonlinear association between hazard for death and tumor volume (*p* = 0.0064). ECOG PS, Eastern Cooperative Oncology Group performance status.

In the multivariable Cox regression analysis, there was a statistically significant association between TV and OS (*p* = 0.0031), ECOG PS and OS (*p* = 0.0002), and receipt of second-line therapy (*p* = 0.0457) (Fig. 1*A* and *B*). The association between TV and OS was nonlinear. The hazard increased as TV increased but plateaued when TV was more than 600 cc. For each 30 cc increase in TV for TV less than 150, the hazard ratio (HR) was approximately 1.1. As TV increased, the HR decreased in size for each 30 cc increase, but there were still significant increases in hazard up to a TV of 300 cc (Table 2). The associations between ECOG PS and OS and receipt of second-line therapy and OS were linear. For each point increase of ECOG PS, the HR was 1.707 (95% confidence interval [CI] [1.275, 2.286]). There were no statistically significant associations between PFS and TV or PFS and ECOG PS. There were no statistically significant associations between response to initial treatment or CTFI (≥6 mo) and OS (*p* = 0.1952 and *p* = 0.1903, respectively). In addition, a logistical regression was performed to find whether patients with lower tumor burdens or lower ECOG PS were more likely to receive second-line therapy, and there were no associations found between these variables (*p* = 0.4631 and *p* = 0.3924, respectively).

**Table 2:** Hazard Ratios for Various Tumor Volumes and Reference Tumor Volumes

Tumor Volume (cc)	Reference Tumor Volume (cc)	HR	95% CI HR	*p* Value
30	0	1.122	[1.036, 1.216]	0.005
60	30	1.119	[1.035, 1.210]	0.005
90	60	1.113	[1.034, 1.198]	0.004
120	90	1.105	[1.032, 1.184]	0.004
150	120	1.098	[1.031, 1.170]	0.004
180	150	1.091	[1.029, 1.157]	0.003
210	180	1.084	[1.028, 1.144]	0.003
240	210	1.078	[1.026, 1.132]	0.003
270	240	1.072	[1.025, 1.120]	0.002
300	270	1.066	[1.023, 1.110]	0.002
630	600	1.014	[0.996, 1.032]	0.130
830	800	0.995	[0.970, 1.021]	0.697

CI, confidence interval; HR, hazard ratio.

## Discussion

This study evaluated the relationship between TV at time of first relapse and clinical outcomes in relapsed SCLC. In this study’s patient population, the mean TV of 199.99 cc was higher than the mean TV of 22.54 cc reported in the paper by Smith et al.^6^ evaluating the relationship between TV and ctDNA. The data set of this study includes tumor burden higher than that of any other study found in the literature review. The near majority of TV in this cohort fell within the 0 to 60 cc range (60 cc is the equivalent to a tumor sphere with a diameter of 4.8 cm). This suggests that at time of relapse, many tumors are already substantial in size. This raises the possibility that earlier detection of relapse could allow for intervention at lower tumor burdens, potentially improving clinical outcomes.

Regarding PFS, this study did not find any statistically significant associations between PFS and ECOG PS and TV. It is not surprising that neither ECOG PS nor TV was statistically associated with PFS as multiple other studies analyzing clinical outcomes in relapsed SCLC have also had mixed results.^7^, ^8^, ^9^, ^10^

The most notable and provoking finding in this study was the statistically significant association between TV and OS. Although lead time bias and confounding may be contributing to this association, this warrants further investigation.^9^ If the relationship between TV and OS is not fully explained by lead time bias or confounding, this may suggest that earlier detection and treatment of relapsed of SCLC, when TV is still lower, may improve survival outcomes.

It was also notable that receipt of second-line therapy was associated with improved OS, but that CTFI and response to initial therapy were not significantly associated with improved OS. The prediction was that all three would be associated with OS, so it was not surprising that receipt of second-line therapy was associated with improved OS. Response to initial therapy likely was not statistically significantly associated with OS due to the lack of variability in response. Two patients had complete response, 79 patients (85%) had partial response, three patients had mixed response, nine patients had stable disease, and one patient had progression of disease. If there were more patients in the complete response, stable disease, and progression of disease categories, a statistically significant association may have been detected. Although previous studies have demonstrated an association between CTFI and OS, the analysis did not confirm this relationship. This discrepancy may reflect differences in patient characteristics or treatment patterns. In addition, the small sample size and relatively small proportion of patients in the CTFI more than or equal to 6 months may have affected the ability to detect a significant association.

## Limitations and Future Directions

This study is inherently retrospective and limited by its modest sample size. In addition, as noted previously, the multivariable Cox regression limited the variables to TV, ECOG PS, response to initial therapy, CTFI, and receipt of second-line therapy to maximize statistical validity at the expense of accounting for other potential confounders. Other potential confounding variables to account for would be demographic and clinical factors (age, sex, initial stage of disease, time between relapse and start of next line of therapy), disease burden variables (number and location of metastatic sites initially and at time of relapse), and nutritional and laboratory markers (weight loss at relapse, serum lactate dehydrogenase, carcinoembryonic antigen, vascular endothelial growth factor, hemoglobin, albumin, sodium, and neutrophil-to-lymphocyte ratio). However, when choosing which additional factors to include, future studies must prioritize key variables and avoid incorporating multiple highly correlated variables.

Another limitation to note in this study is the heterogeneity in second-line therapies that patients received. This certainly could have affected the strength of association between TV and OS found in this analysis. As more research is done to identify superior second-line treatments, patients with relapsed SCLC may receive more homogenous second-line treatments, and this analysis could be repeated to see whether the strength of association is changed. Alternatively, future studies could use inclusion criteria of CTFI more than 6 months, as the second-line therapy is generally platinum doublet rechallenge, which would result in a cohort with more homogeneity in second-line therapy.

Another important limitation to note is that TV was only measured at time of first relapse. It would have been ideal to also have measured TV at time of first diagnosis. Unfortunately, due to the time-consuming nature of measuring TV, it was done at only one time point, and the time of first relapse was chosen because it was the most relevant.

The results of this study suggest the feasibility of increasing the frequency of surveillance CT scans as an avenue to improving clinical outcomes in relapsed SCLC. However, the costs of increased surveillance must also be considered. Increasing frequency of surveillance CT scans could lead to earlier detection and treatment of relapse, but it could come with the harm of increased financial costs, time burdens, stress, and anxiety for patients, which may negatively affect survival. In the case of relapsed SCLC, because clinical outcomes are so poor, the side effects of increased radiation from more frequent scans would likely be insignificant.

Future directions could include the use of ctDNA as a means of detecting relapse before there is radiographic evidence of relapse, as research has revealed associations between ctDNA and TV.^6^ Furthermore, in patients who have received radiotherapy, it may be difficult to distinguish pseudo-progression from true progression, so ctDNA could be a helpful marker to detect relapse especially in that scenario.

In conclusion, this study found a statistically significant association between TV and OS in patients with relapsed SCLC. Future studies should aim to evaluate whether initiation of standard systemic therapy at lower TV can consistently improve PFS and OS. Future studies should also aim to evaluate the use of ctDNA as a means of detecting and treating relapsed SCLC even earlier to improve clinical outcomes.

## CRediT Authorship Contribution Statement

**Matthew Lu:** Methodology, Investigation, Data curation, Writing - original draft and final revisions, Visualization, Project administration.

**Hayden Byrd:** Software, Formal analysis, Writing - original draft.

**Heidi Chen:** Software, Formal analysis, Visualization, Writing - original draft.

**Wade Iams:** Conceptualization, Data curation, Writing - review & editing, Supervision.

## Disclosure

Dr. Iams reported serving as a consultant for OncLive, Clinical Care Options, Chardan, Cello Health, and Curio Science. Dr. Iams also served on the advisory boards for Genentech, Mirati, Outcomes Insights, Jazz Pharmaceuticals, GI Therapeutics, Takeda, AstraZeneca, Sanofi, Janssen, Amgen, Bristol Myers Squibb, and NovoCure. The remaining authors declare no conflict of interest.
